# Community-Level Breast Cancer Screening Estimates, Alaska, 2014

**DOI:** 10.5888/pcd16.180654

**Published:** 2019-04-25

**Authors:** Abigail Newby-Kew, Cheley Grigsby, Charles J. Utermohle

**Affiliations:** 1 Alaska Division of Public Health, Section of Women’s, Children’s, and Family Health, Anchorage, Alaska; 2 Alaska Division of Public Health, Section of Chronic Disease Prevention and Health Promotion, Anchorage, Alaska

**Figure:**
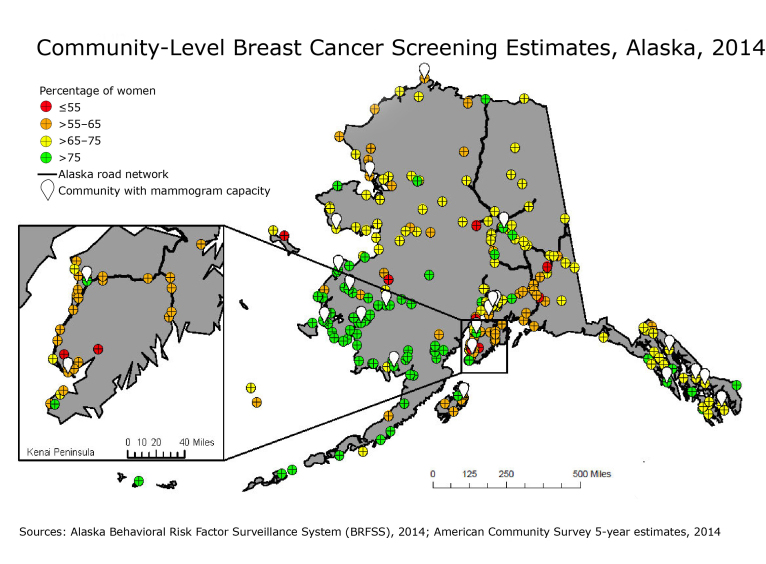
Estimated percentage of women aged 45 to 74 in Alaska who reported receiving a mammogram in the previous 2 years, based on the 2014 Behavioral Risk Factor Surveillance System and the 2014 American Community Survey (ACS) 5-year estimates. A community was defined as any city, town, or unique rural population center. Communities with mammogram capability may be only partially staffed. This map helped the Alaska Breast Cancer Coalition prioritize screening outreach efforts.

## Background

Breast cancer is the most commonly occurring cancer among women in Alaska, and the second leading cause of cancer-related mortality (1). Regular screening with mammography is associated with reduced breast cancer mortality (2). Nationally, women who are uninsured, have a low level of education, or have a low household income are less likely to report having had a mammogram in the previous 2 years (3). Annual breast cancer screening rates in Alaska have consistently been below the US median during the past decade; in 2014, 68% of Alaska women aged 50 to 74, compared with 76% of US women in the same age group, reported having had a mammogram in the past 2 years (4).

Through the National Breast and Cervical Cancer Early Detection Program (NBCCEDP), the Centers for Disease Control and Prevention (CDC) funds 5 organizations in Alaska, including the Division of Public Health and 4 tribal agencies. These grantees provide breast cancer screening and diagnostic services for low-income, uninsured, and underserved women in Alaska. Alaska’s approximately 750,000 residents are dispersed among more than 350 communities, most of which are not on the state’s road system (5). Each community is unique in demographic characteristics, local health services, and ease of access to resources in larger hub communities. Twenty-one communities have mammography capabilities, and 2 mobile mammography units visit communities along the road and ferry network.

The Behavioral Risk Factor Surveillance System (BRFSS) is the primary source for estimates of cancer screening rates in Alaska. These estimates are invaluable for tracking screening rates over time; however, because of the survey’s sampling structure, regional rates are the most granular geographic estimates that it can generate. The Alaska Breast Cancer Coalition conducted a geographic information system (GIS) analysis to fulfill the programmatic need for community-level breast cancer screening estimates and to inform NBCCEDP community-level outreach efforts and interventions.

## Data Sources and Map Logistics

We obtained data from the 2014 Alaska BRFSS on the percentage of women aged 45 to 74 who reported having had a mammogram in the previous 2 years. We stratified the data by household income relative to the federal poverty level (FPL) and the 7 Alaska public health regions (6). We obtained data on the number of women aged 45 to 74 at or above and below the FPL from the US Census Bureau American Community Survey (ACS) 5-year estimates, 2010 through 2014. These data are provided for incorporated cities and census-designated places (CDPs), unincorporated population centers that are identifiable by name. Cities and CDPs correspond to community boundaries in Alaska, and regional delineations do not cross city or CDP boundaries.

For the 2 economic strata of interest, 1) at or above the FPL and 2) below the FPL, we estimated the number of women screened in each community by applying the regional BRFSS screening rates to the ACS community-level counts of eligible women. We then calculated the community-level screening rate by summing the estimated number of screened women in the 2 economic strata and dividing the total number screened by the total age-eligible population:

We classified community screening rates into 4 categories, chosen to visualize differences within regions and between regions (<55%, >55% to 65%, >65% to 75%, and >75%) and plotted these communities and categories on a map of Alaska. Analyses were conducted in SAS version 9.4 (SAS Institute, Inc) and mapped in ArcGIS Desktop version 10.2 (Esri).

## Highlights

The community-level screening estimates ranged from 45.5% to 90.2%. The map was useful for NBCCDEP grantees and the Alaska Breast Cancer Coalition to understand potential differences in screening rates among the communities they support, particularly communities in the same region. One limitation of our analysis was that we applied regional rates to communities with low population counts, so estimates for these communities may not be reliable.

An unexpected finding was the low estimated screening rates on the Kenai Peninsula (inset map), which ranged from 53.5% to 77.2%. Communities on the Kenai Peninsula are on the road network and are near 2 permanent mammography centers. The Alaska Breast Cancer Coalition had assumed that this access to services would lead to high screenings rates in this region. Our analysis suggested that women and/or providers on the Kenai Peninsula may have misconceptions about mammograms or may lack awareness about current screening recommendations.

## Action

On the basis of this analysis, the Alaska Breast Cancer Coalition prioritized screening outreach efforts on the Kenai Peninsula. Coalition members conducted a community survey and key informant interviews to assess attitudes about breast cancer screening and potential barriers, which informed the development of tailored educational materials. They also coordinated with the mobile mammography units to ensure regional coverage and boosted outreach and advertising before events. They continue to foster partnerships with community stakeholders to identify new strategies to increase screening and will closely monitor data through a future GIS analysis of community-level changes.

## References

[1] Alaska Cancer Registry. Cancer in Alaska: multi-year summary report, 2017. Anchorage (AK): Section of Chronic Disease Prevention and Health Promotion, Division of Public Health, Alaska Department of Health and Social Services; 2017.

[2] Myers ER , Moorman P , Gierisch JM , Havrilesky LJ , Grimm LJ , Ghate S , Benefits and harms of breast cancer screening: a systematic review. JAMA 2015;314(15):1615–34. 10.1001/jama.2015.13183 26501537

[3] White A , Thompson TD , White MC , Sabatino SA , de Moor J , Doria-Rose PV , Cancer screening test use — United States, 2015. MMWR Morb Mortal Wkly Rep 2017;66(8):201–6. 10.15585/mmwr.mm6608a1 28253225 PMC5657895

[4] Indicator-Based Information System for Public Health (AK-IBIS). Anchorage (AK): Alaska Department of Health and Social Services. http://ibis.dhss.alaska.gov/. Accessed May 1, 2018.

[5] Alaska population overview 2016 estimates. Anchorage (AK): Department of Labor and Workforce Development, Research and Analysis Section; 2017. http://live.laborstats.alaska.gov/pop/estimates/pub/16popover.pdf. Accessed February 11, 2019.

[6] Informed Alaskans. Alaska health profiles geography: public health regions. Anchorage (AK): Alaska Department of Health and Social Services; 2018. http://dhss.alaska.gov/dph/InfoCenter/Pages/ia/geo_phr.aspx. Accessed May 3, 2018.

